# Supplementary material for: In Vitro Reconstitution of Vertebrate Sonic Hedgehog Protein Cholesterolysis; Native and Engineered Sterol Specificity
Source: Biochemistry. 2026 Oct 13;65(21):3533–47. doi: 10.1021/acs.biochem.6c00321 (PMC13738340; doi:10.1021/acs.biochem.6c00321)
Supplement: Supplementary file 1 [file bi6c00321_si_001.pdf]

Supporting Information

In vitro reconstitution of vertebrate Sonic Hedgehog protein cholesterolysis; native and engineered sterol specificity

Dayton C. Seidel[1‡], Andrew G. Wagner[1‡], John L. Pezzullo[2], Katherine A. Thayer[2], Seth Beadle[2], Margot L. Olejarczyk[2], Logan R. Bartlett[1], José-Luis Giner[2*], Brian P. Callahan[1*]

[1]Department of Chemistry, Binghamton University, State University of New York, Binghamton, New York 13902, United States

[2]Department of Chemistry, State University of New York College of Environmental Science and Forestry, Syracuse, New York 13210, United States

‡ Equal contribution

*Corresponding Authors: Brian P. Callahan — Department of Chemistry, Binghamton University, State University of New York, Binghamton, New York 13902, United States; callahan@binghamton.edu; José-Luis Giner — Department of Chemistry, State University of New York College of Environmental Science and Forestry, Syracuse, New York 13210, United States; jlginer@syr.edu

Supporting Tables 1-6 .... S2-S7

Figure S1 Sequence alignment of Dme, Xla, Dre, and Homo Sapiens SHhC S8

Figure S2 Amino acid sequences of Dme, Dre, and Xla C-H-Y S9

Figure S3 SDS-PAGE of bacterially expressed Dme, Dre, Xla C-H-Y reporter constructs S10

Figure S4 Wild-type C-H-Y reporter constructs expressed in LMG194 vs. Bl21-AI E. coli strains S11

Figure S5 Observed FRET of Dme, Dre, and Xla C-H-Y constructs S12

Figure S6 C1A point mutant eliminates autoprocessing activity S13

Figure S7 Rates of Thiolysis plotted as a function of increasing DTT concentration S14

Figure S8 Key for the 96-well detergent screen S15

Figure S9 Impact of Fos-Choline 12 concentration on FRET signal from C-H-Y S16

Figure S10 Apparent species-specific detergent effects S17

Figure S11 SDS-PAGE Analysis of substrate activity for chemical rescue sterols S18

Figure S12 Substrate activity of synthetic sterols using Wild-Type C-H-Y reporter constructs S19

Figure S13 Substrate activity of the 2-beta carboxy cholestanol epimer S20

Figure S14 Concentration-dependent activity of the 2-beta carboxy cholestanol epimer S21

Figure S15 Substrate activity of cholestanol 2-imidazolmethyl epimers S22

References S23

Supporting Tables

Table S1. UniProt residue numbers corresponding to the C1 and D46 positions in HhC in all species constructs[a]

	Dme HhC	Dre SHhaC	Xla SHhC
C1 residue position	258	198	199
D46 residue position	303	243	244

[a] UniProt accession numbers: Q02936 (Dme), Q92008 (Dre), Q92000 (Xla), Q15465 (*Homo Sapiens*).

Table S2. Sequence identity and similarity of the cholesterolysis domains from model organisms compared to human SHhC

	Dme HhC	Dre SHhaC	Xla SHhC
Identity	32%	46%	49%
Similarity	45%	60%	59%

Table S3. Apparent rates of spontaneous precursor hydrolysis at 30 °C, pH 7.1, for *Drosophila* (Dme), *Danio* (Dre), and *Xenopus* (Xla) Wild-Type and D46A SHhC using the FRET reporter C-H-Y

Construct	k_{obs} (s^{-1})	$t_{1/2}$ (hrs)
Dme HhC (WT)	$0.58*10^{-5} \pm 0.16*10^{-5}$	33
Dre SHhaC (WT)	$4.3*10^{-5} \pm 1.2*10^{-5}$	4.5
Xla SHhC (WT)	$4.2*10^{-5} \pm 2.9*10^{-5}$	4.6
Dme HhC (D46A)	$0.66*10^{-5} \pm 0.15*10^{-5}$	29
Dre SHhaC (D46A)	$5.3*10^{-5} \pm 0.79*10^{-5}$	3.6
Xla SHhC (D46A)	$4.2*10^{-5} \pm 0.94*10^{-5}$	4.6

Rate constants and confidence intervals are estimated based on extrapolated FRET-loss trajectories. Reactions did not reach completion during the assay period. Individual rate constants and averaged values were obtained using the solver function in Excel with 95% confidence intervals generated from the descriptive statistics function (n=4).

Table S4. Thiolysis activity with Dithiothreitol (DTT) for Dme, Dre, and Xla WT and D46A SHhC using FRET reporter[a]

	k_{obs} (s^{-1})			
Construct	100 mM DTT	50 mM DTT	5 mM DTT	1 mM DTT
Dme HhC (WT)	$6.0*10^{-4} \pm 0.29*10^{-4}$	$3.4*10^{-4} \pm 0.09*10^{-4}$	[b] $0.53*10^{-4} \pm 0.09*10^{-4}$	[b] $0.13 \times 10^{-4} \pm 0.06*10^{-4}$
Dre SHhaC (WT)	$21*10^{-4} \pm 1.1*10^{-4}$	$15*10^{-4} \pm 0.75*10^{-4}$	$3.4*10^{-4} \pm 0.17*10^{-4}$	[b] $0.93*10^{-4} \pm 0.02*10^{-4}$
Xla SHhC (WT)	$12*10^{-4} \pm 2.5*10^{-4}$	$6.9*10^{-4} \pm 0.50*10^{-4}$	[b] $1.2*10^{-4} \pm 0.49*10^{-4}$	[b] $0.23*10^{-4} \pm 0.08*10^{-4}$
Dme HhC (D46A)	$13*10^{-4} \pm 1.7*10^{-4}$	$9.0*10^{-4} \pm 0.90*10^{-4}$	$1.4*10^{-4} \pm 0.30*10^{-4}$	[b] $0.30*10^{-4} \pm 0.25*10^{-4}$
Dre SHhaC (D46A)	$28*10^{-4} \pm 1.5*10^{-4}$	$22*10^{-4} \pm 0.84*10^{-4}$	$11*10^{-4} \pm 0.28*10^{-4}$	$2.9*10^{-4} \pm 0.80*10^{-4}$
Xla SHhC (D46A)	$23*10^{-4} \pm 1.2*10^{-4}$	$16*10^{-4} \pm 0.61*10^{-4}$	$5.5*10^{-4} \pm 1.3*10^{-4}$	[b] $0.85*10^{-4} \pm 0.33*10^{-4}$

Confidence intervals generated using the Excel descriptive statistics function (n=3).

[a] Rate constants and averaged values were obtained using the solver function in Excel.

[b] Confidence intervals are estimated based on data from reactions that did not reach completion

Table S5. Compatible detergents for cholesterolysis with Dme, Dre, and Xla SHhC at 30 ^oC, in Bis-Tris buffer (pH 7.1)[a]

Dme			Dre			Xla		
Detergent[b]	Type	k_{obs} (s^{-1})	Detergent[b]	Type	k_{obs} (s^{-1})	Detergent[b]	Type	k_{obs} (s^{-1})
14:0 Lyso PG	I	0.79*10^{-3}	14:0 Lyso PG	I	0.56*10^{-3}	Brij 35	N	0.89*10^{-3}
18:0 Lyso PG	I	0.65*10^{-3}	18:1 Lyso PG	I	0.65*10^{-3}	Brij 56	N	0.82*10^{-3}
Brij 35	N	0.69*10^{-3}	APO 12	N	0.50*10^{-3}	Brij 58	N	0.89*10^{-3}
Genapol X-080	N	0.80*10^{-3}	Brij 56	N	0.65*10^{-3}	Genapol X-080	N	0.85*10^{-3}
n-Decanoylsucrose	N	0.89*10^{-3}	Brij 58	N	0.51*10^{-3}	DDAO	N	0.61*10^{-3}
Nonylphenyl polyethylene glycol	N	0.95*10^{-3}	Genapol X-080	N	0.84*10^{-3}	n-Decanoylsucrose	N	0.68*10^{-3}
10:0 Lyso PC	Z	0.73*10^{-3}	Mal(11.2)	N	0.62*10^{-3}	Nonylphenyl polyethylene glycol	N	1.0*10^{-3}
12:0 Lyso PC	Z	1.2*10^{-3}	n-Decyl-β-D-maltoside	N	0.65*10^{-3}	Tween 20	N	1.0*10^{-3}
13:0 Lyso PC	Z	0.95*10^{-3}	n-Decanoylsucrose	N	0.63*10^{-3}	Tween 80	N	1.0*10^{-3}
15:0 Lyso PC	Z	0.93*10^{-3}	N-Nonyl-β-D-glucoside	N	0.74*10^{-3}	Zwittergent 3-16	N	0.82*10^{-3}
Fos-Choline 10	Z	0.66*10^{-3}	Nonylphenyl polyethylene glycol	N	0.71*10^{-3}	10:0 Lyso PC	Z	0.73*10^{-3}
**Fos-Choline 12**	**Z**	**1.9*10^{-3}**	Tween 20	N	0.82*10^{-3}	12:0 Lyso PC	Z	1.3*10^{-3}
Fos-Choline 14	Z	1.3*10^{-3}	Zwittergent 3-16	N	1.0*10^{-3}	13:0 Lyso PC	Z	1.0*10^{-3}
Fos-Choline 16	Z	1.0*10^{-3}	07:0 PC (DHPC)	Z	1.1*10^{-3}	15:0 Lyso PC	Z	0.86*10^{-3}

[a] Values reported were calculated using the solver function in Excel (n=1).

[b] Detergents plate locations can be found in **Supporting Figure 8**

Table S5 Continued.

Dre			Xla		
Detergent[b]	Type	k_{obs} (s^{-1})	Detergent[b]	Type	k_{obs} (s^{-1})
12:0 Lyso PC	Z	$0.98*10^{-3}$	17:0 Lyso PC	Z	$1.1*10^{-3}$
13:0 Lyso PC	Z	$0.88*10^{-3}$	18:0 Lyso PC	Z	$1.3*10^{-3}$
15:0 Lyso PC	Z	$0.80*10^{-3}$	18:1 Lyso PC	Z	$1.1*10^{-3}$
18:0 Lyso PC	Z	$0.84*10^{-3}$	Fos-Choline 10	Z	$0.99*10^{-3}$
18:1 Lyso PC	Z	$1.2*10^{-3}$	**Fos-Choline 12**	**Z**	$\mathbf{1.6*10^{-3}}$
**Fos-Choline 12**	**Z**	$\mathbf{1.4*10^{-3}}$	Fos-Choline 14	Z	$0.95*10^{-3}$
Fos-Choline 14	Z	$0.93*10^{-3}$	Fos-Choline 16	Z	$1.1*10^{-3}$

[b] Detergents plate locations can be found in **Supporting Figure 8**

Table S6. Substrate activity of engineered sterols with Dme, Dre, and Xla Wild-Type HhC[a]

Substrate	Structure	k_{obs} (s^{-1})		
		Dme HhC	Dre SHhaC	Xla SHhC
3-AOCp	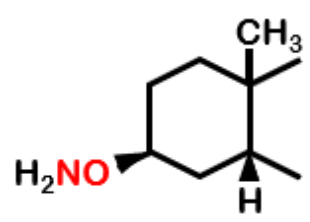	$0.71*10^{-3} \pm 0.26*10^{-3}$	[b] $\leq 4.3*10^{-5} \pm 1.2*10^{-5}$	[b] $8.6*10^{-5} \pm 5.9*10^{-5}$
3-HPC	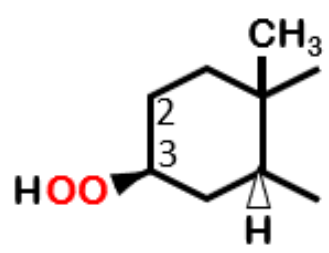	$1.1*10^{-3} \pm 0.18*10^{-3}$	$0.67*10^{-3} \pm 0.06*10^{-3}$	$0.52*10^{-3} \pm 0.12*10^{-3}$
2-AMI	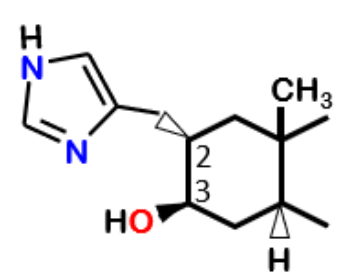	$0.82*10^{-3} \pm 0.13*10^{-3}$	[b] $0.3*10^{-3} \pm 0.09*10^{-3}$	$0.49*10^{-3} \pm 0.08*10^{-3}$
2-ACC	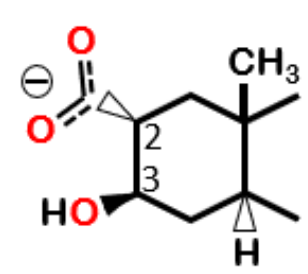	$2.9*10^{-3} \pm 0.59*10^{-3}$	$1.8*10^{-3} \pm 0.40*10^{-3}$	$1.6*10^{-3} \pm 0.24*10^{-4}$
epi 2-ACC	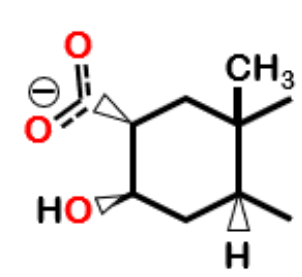	[b] $3.4*10^{-5} \pm 1.6*10^{-5}$	[b] $\leq 4.3*10^{-5} \pm 1.2*10^{-5}$	[b] $\leq 4.2*10^{-5} \pm 2.9*10^{-5}$
2-BCC	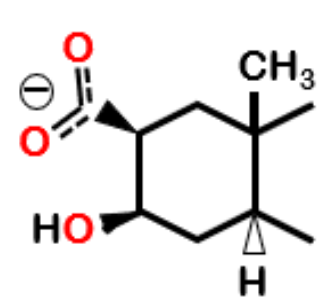	[b] $2.8*10^{-5} \pm 0.82*10^{-5}$	[b] $\leq 4.3*10^{-5} \pm 1.2*10^{-5}$	[b] $\leq 4.2*10^{-5} \pm 2.9*10^{-5}$

[a] Rate constants and their averages were calculated using the solver function in Excel with confidence intervals generated using the descriptive statistics function (n=3).

[b] Rate constants and confidence intervals are estimated based on data from reactions that did not reach completion during the assay interval.

Supporting Figures

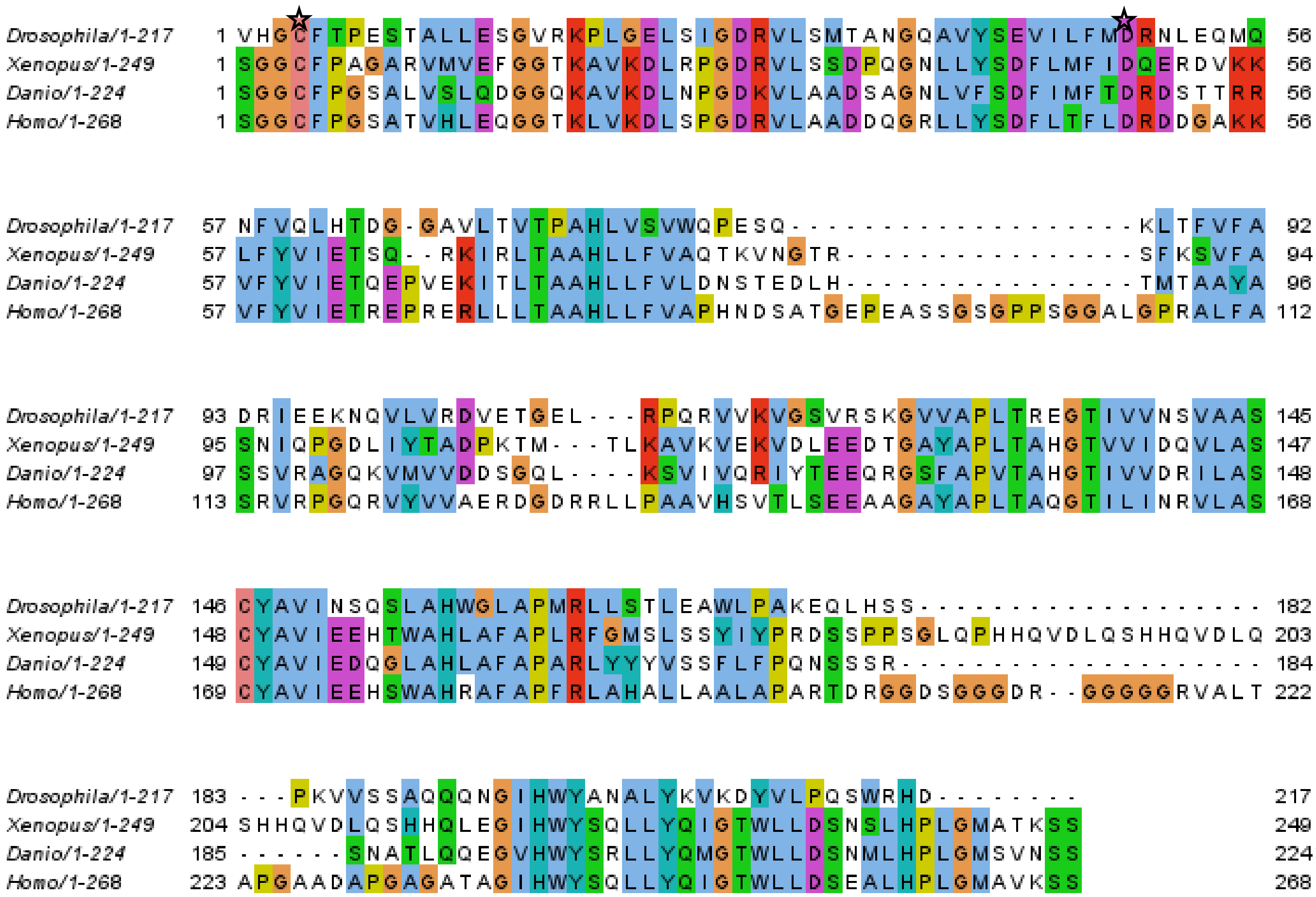

Figure S1. Sequence alignment of Dme, Xla, Dre, and *Homo Sapiens* SHhC. Uniprot accession numbers: Q02936 (Dme), Q92008 (Dre), Q92000 (Xla), Q15465 (*Homo Sapiens*). The identity and similarity values are summarized in **supporting table 1**. The catalytically essential C1 and D46 residues are indicated with a star. Note that the (-3) residue here is numbered as (1).

Amino acid sequence of Dme C-H-Y:

MSKGEELFGGIVPILVELEGDVNGHKFSVSGEGEGDATYGKLTLKFICTTGKLPVPWPTLVTTLTWGVQC
FSRYPDHMKQHDFFKSVMPEGYVQERTIFFKDDGNYKTRAEVKFEGDTLVNRIELKGIDFKEDGNILGHK
LEYNYISHNVYITADKQKNGIKANFKARHNITDGSVQLADHYQQNTPIGDGPVILPDNHYLSTQSALSKDP
NEKRDHMVLLEFVTAAGITHGMDELYKGSGGSTVHG**C**FTPESTALLESGVRKPLGELSIGDRVLSMTAN
GQAVYSEVILFM**D**RNLEQMQNFVQLHTDGGAVLTVTPAHLVSVWQPESQKLTFVFADRIEEKNQVLVRD
VETGELRPQRVVKVGSVRSKGVVAPLTREGTIVVNSVAASCYAVINSQSLAHWGLAPMRLLSTLEAWLPA
KEQLHSSPKVVSSAQQQNGIHWYANALYKVKDYVLPQSWRHDGGLQGSGGSMSKGEELFTGVVPILVE
LDGDVNGHKFSVSGEGEGDATYGKLTLKLLCTTGKLPVPWPTLVTTLGYGVQCFARYPDHMKQHDFFK
SAMPEGYVQERTIFFKDDGNYKTRAEVKFEGDTLVNRIELKGIDFKEDGNILGHKLEYNYNSHNVYITADK
QKNGIKANFKIRHNIEDGGVQLADHYQQNTPIGDGPVLLPDNHYLSYQSALFKDPNEKRDHMVLLEFLTA
AGITEGMNELYKHHHHHH

Amino acid sequence of Dre C-H-Y:

MSKGEELFGGIVPILVELEGDVNGHKFSVSGEGEGDATYGKLTLKFICTTGKLPVPWPTLVTTLTWGVQC
FSRYPDHMKQHDFFKSVMPEGYVQERTIFFKDDGNYKTRAEVKFEGDTLVNRIELKGIDFKEDGNILGHK
LEYNYISHNVYITADKQKNGIKANFKARHNITDGSVQLADHYQQNTPIGDGPVILPDNHYLSTQSALSKDP
NEKRDHMVLLEFVTAAGITHGMDELYKGSGGSRVHGG**C**FPGSALVSLQDGGQKAVKDLNPGDKVLAAD
SAGNLVFSDFIMFT**D**RDSTTRRVFYVIETQEPVEKITLTAAHLLFVLDNSTEDLHTMTAAYASSVRAGQKV
MVVDDSGQLKSVIVQRIYTEEQRGSFAPVTAHGTIVVDRILASCYAVIEDQGLAHLAFAPARLYYYVSSFLF
PQNSSSRSNATLQQEGVHWYSRLLYQMGTWLLDSNMLHPLGMSVNSSGGLQGSGGSMSKGEELFTG
VVPILVELDGDVNGHKFSVSGEGEGDATYGKLTLKLLCTTGKLPVPWPTLVTTLGYGVQCFARYPDHMK
QHDFFKSAMPEGYVQERTIFFKDDGNYKTRAEVKFEGDTLVNRIELKGIDFKEDGNILGHKLEYNYNSHN
VYITADKQKNGIKANFKIRHNIEDGGVQLADHYQQNTPIGDGPVLLPDNHYLSYQSALFKDPNEKRDHMV
LLEFLTAAGITEGMNELYKHHHHHH

Amino acid sequence of Xla C-H-Y:

MSKGEELFGGIVPILVELEGDVNGHKFSVSGEGEGDATYGKLTLKFICTTGKLPVPWPTLVTTLTWGVQC
FSRYPDHMKQHDFFKSVMPEGYVQERTIFFKDDGNYKTRAEVKFEGDTLVNRIELKGIDFKEDGNILGHK
LEYNYISHNVYITADKQKNGIKANFKARHNITDGSVQLADHYQQNTPIGDGPVILPDNHYLSTQSALSKDP
NEKRDHMVLLEFVTAAGITHGMDELYKGSGGSRSGG**C**FPAGARVMVEFGGTKAVKDLRPGDRVLSSDP
QGNLLYSDFLMFI**D**QERDVKKLFYVIETSQRKIRLTAAHLLFVAQTKVNGTRSFKSVFASNIQPGDLIYTAD
PKTMTLKAVKVEKVDLEEDTGAYAPLTAHGTVVIDQVLASCYAVIEEHTWAHLAFAPLRFGMSLSSYIYPR
DSSPPSGLQPHHQVDLQSHHQVDLQSHHQVDLQSHHQLEGIHWYSQLLYQIGTWLLDSNSLHPLGMAT
KSSLQGSGGSMSKGEELFTGVVPILVELDGDVNGHKFSVSGEGEGDATYGKLTLKLLCTTGKLPVPWPT
LVTTLGYGVQCFARYPDHMKQHDFFKSAMPEGYVQERTIFFKDDGNYKTRAEVKFEGDTLVNRIELKGID
FKEDGNILGHKLEYNYNSHNVYITADKQKNGIKANFKIRHNIEDGGVQLADHYQQNTPIGDGPVLLPDNHY
LSYQSALFKDPNEKRDHMVLLEFLTAAGITEGMNELYKHHHHHH

Figure S2. Amino acid sequences of Dme, Dre, and Xla C-H-Y. Cyan fluorescent protein (cyan); SHhC (gray); yellow fluorescent protein (yellow). The catalytically essential C1 and D46 residues, are labeled in bold. Sequences were verified by whole plasmid sequencing (Azenta).

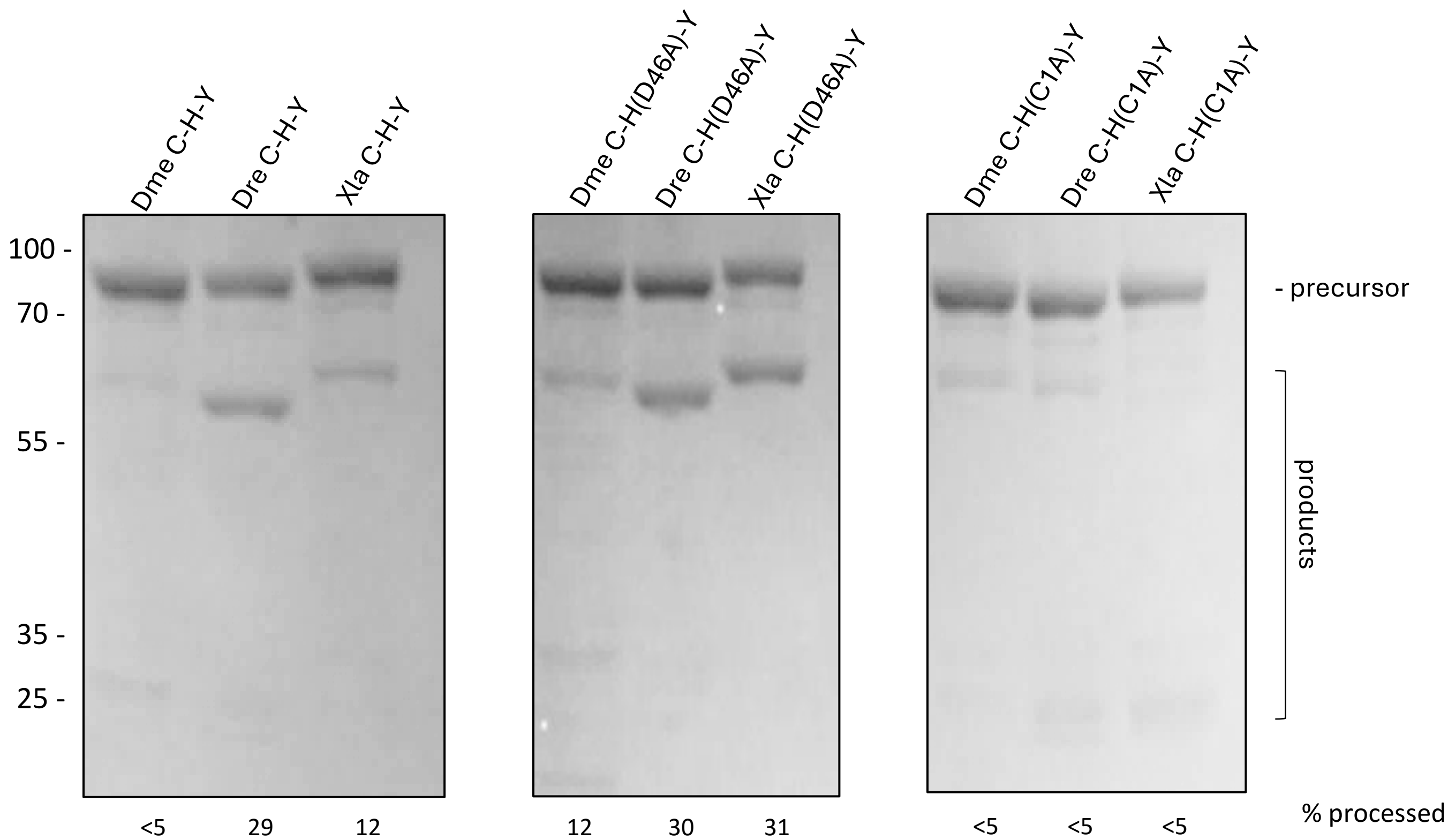

Figure S3. SDS-PAGE of bacterially expressed, Ni-NTA purified Dme, Dre, Xla C-H-Y reporter constructs. The extent of spontaneous precursor autoprocessing that occurred during the E. coli expression and Ni-NTA purification is indicated below each gel. Protein samples were run at 2 μM precursor concentration, and Coomassie stained for visualization. The extent of processing was determined by ImageJ.

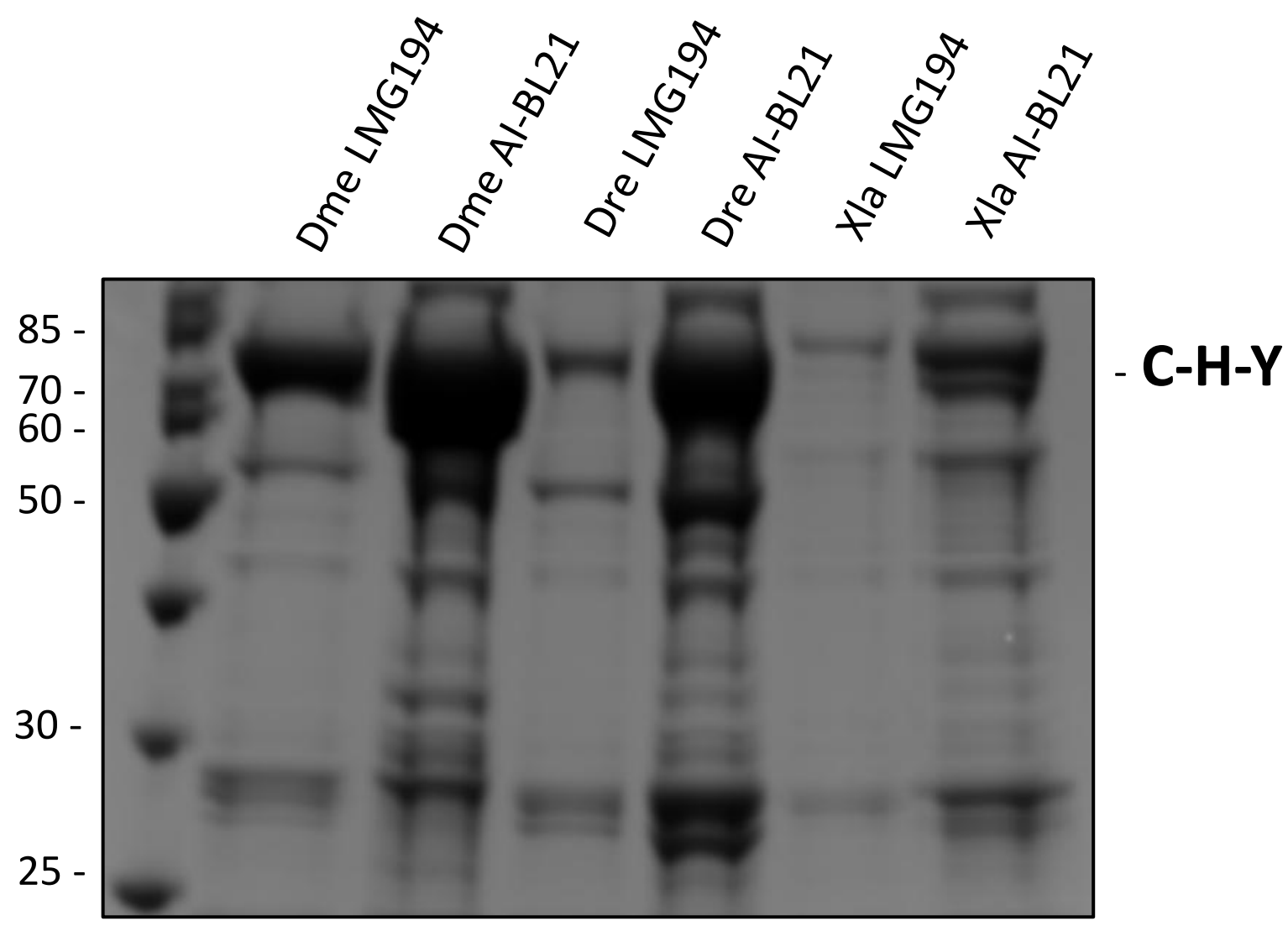

Figure S4. Comparison of Ni-NTA purified Dme, Dre, Xla wild-type C-H-Y reporter constructs expressed in LMG194 vs. BL21-AI E. coli strains. The average fold-change of precursor between the LMG194 and AI-BL21 cells was calculated by image analysis (BioRad Gel Doc EZ system) of the C-H-Y protein bands using a 1 mg/mL BSA sample as the comparator. The average (n=3) fold-increase in going from LMG194 to AI-BL21: 2.7x for Dme C-H-Y, 3.6x for Dre C-H-Y, and 7.3x for Xla C-H-Y. Gels were Coomassie stained for visualization.

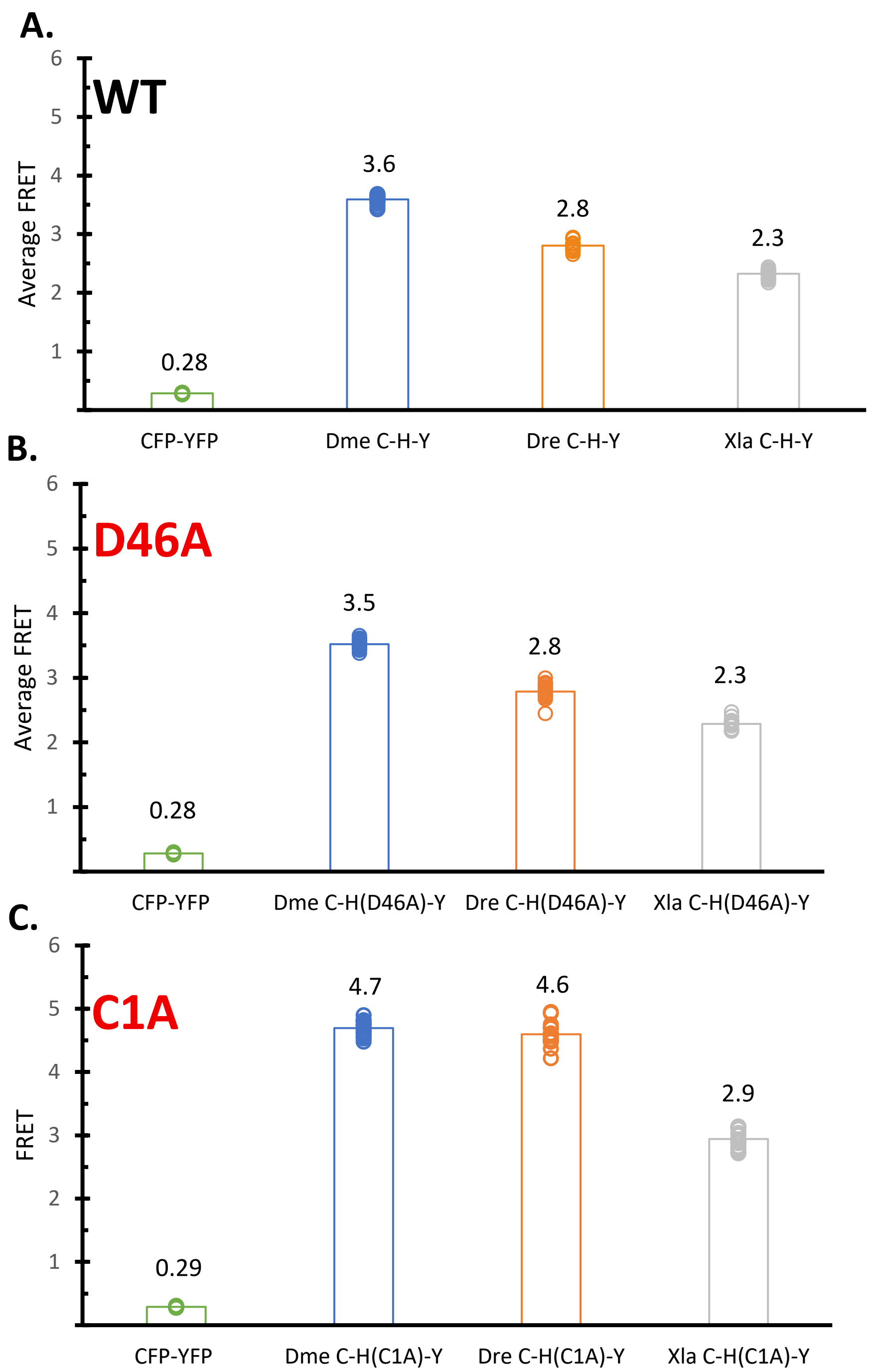

Figure S5. Observed FRET of Dme, Dre, and Xla C-H-Y constructs. Values were recorded at 30°C with 0.2 µM C-H-Y (A), C-H(D46A)-Y (B), or C-H(C1A)-Y (C), Bis-Tris buffer (pH 7.1) containing EDTA (5 mM) and NaCl (0.5 M), and Fos-choline 12 (1.5 mM). CFP-YFP samples contained an equimolar mixture of CFP and YFP under equivalent conditions.

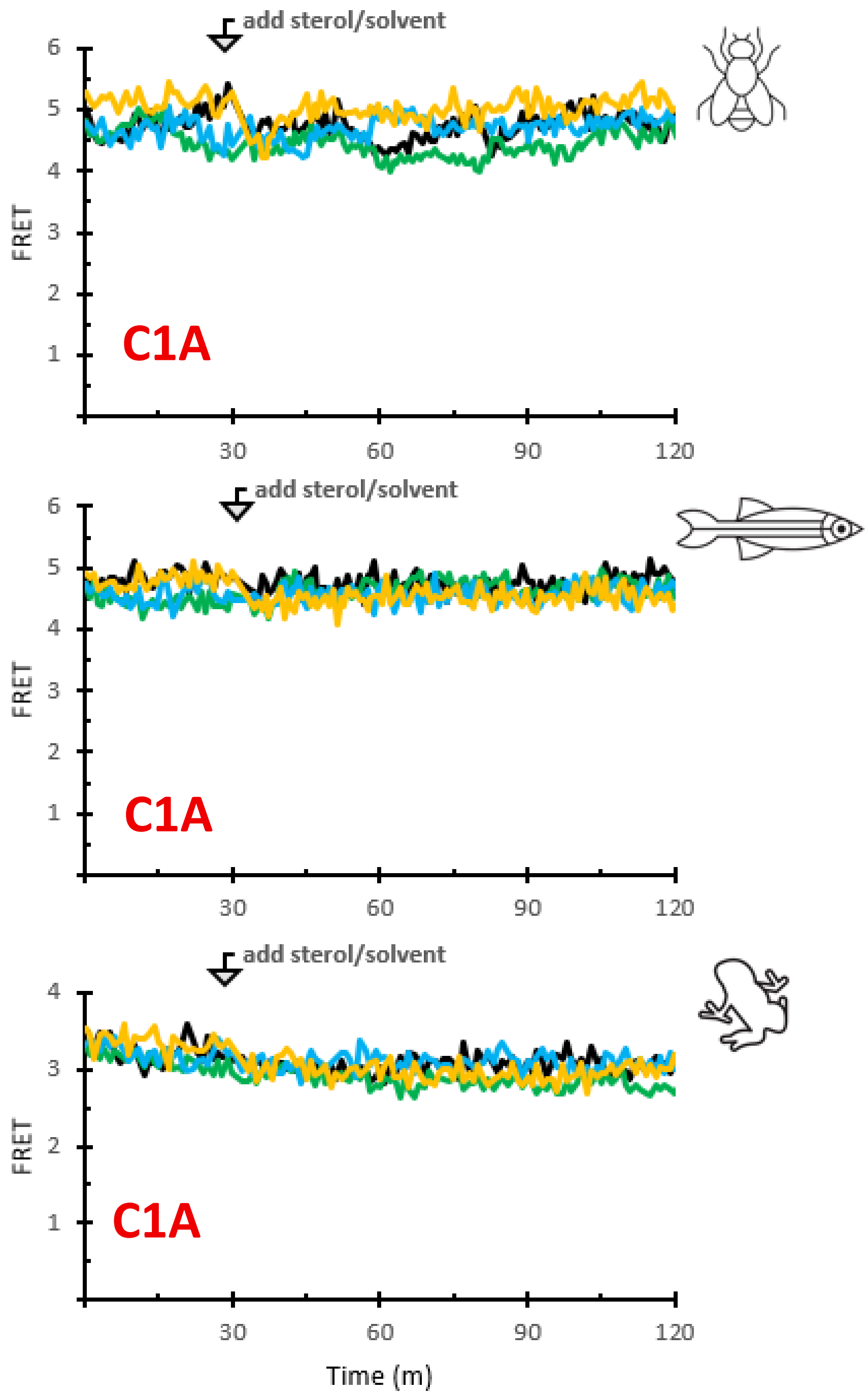

Figure S6. Alanine point mutation at the catalytically essential C1 residue of SHhC eliminates autoprocessing activity. Reactions were run for 2 hours at 30°C in 96-wellplates using 0.2µM C-H(C1A)-Y, Bis-Tris buffer (pH 7.1) containing EDTA (5 mM) and NaCl (0.5 M), and Fos-choline 12 (1.5 mM), with either no sterol (black); 50µM cholesterol (green); 2-ACC (cyan); or 100mM DTT (yellow).

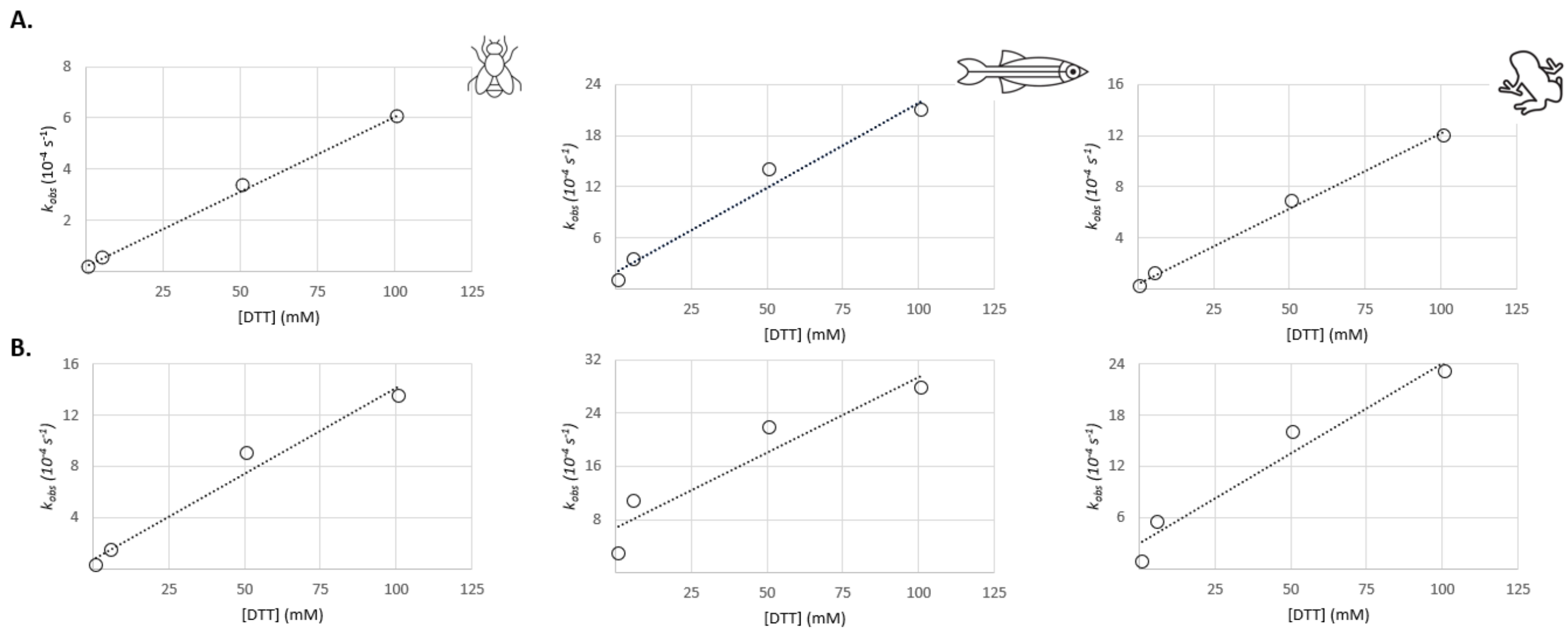

Figure S7. Rates of thioloysis (k_{obs}) plotted as a function of increasing DTT concentration. Reactions were run for 2 hours at 30°C in 96-well plates using 0.2 μM C-H-Y (*A*) or C-H(D46A)-Y (*B*), Bis-Tris buffer (pH 7.1) also containing EDTA (5mM) and NaCl (0.5M), and Fos-choline 12 (1.5mM). (*A*) DTT concentration vs. k_{obs} of DTT-based processing for WT constructs from Dme (left), Dre (middle), or Xla (right). (*B*) DTT concentration vs. k_{obs} as an (A), expect for (D46A) mutants.

	1	2	3	4	5	6	7	8	9	10	11	12
A	14:0 Lyso PG (I) 0.1mM	16:0 Lyso PG (I) 0.6mM	18:0 Lyso PG (I) 0.5mM	18:1 Lyso PG (I) 0.5mM	CTAB (I) 1.0mM	BAM (I) 0.1%w/v	DTAC (I) 0.1mM	Sodium cholate hydrate (I) 15.0mM	Deoxycholic acid sodium salt (I) 6.0mM	MSDH (I) 0.5mM	N-Laurylsarcosine sodium salt (I) 14.6mM	Sodium dodecyl sulfate (I) 10.0mM
B	Lithium dodecyl sulfate (I) 10.0mM	APO 8 (N) 40.0mM	APO 9 (N) 0.5mM	Apo 10 (N) 4.7mM	APO 11 (N) 0.5mM	APO 12 (N) 0.57mM	C_8E_4 (N) 8.0mM	C_8E_5 (N) 7.1mM	$C_{12}E_8$ (N) 0.11mM	$C_{12}E_9$ (N) 0.08mM	Brij 35 (N) 0.1% v/v	Brij 56 (N) 0.1% v/v
C	Brij 58 (N) 0.1% v/v	Genapol X-080 (N) 0.1% v/v	Facade-EM (N) 0.18mM	Facade-EPC (N) 0.97mM	Facade-TEG (N) 0.19mM	Facade-TEM (N) 0.19mM	Facade-TFA1 (N) 0.013mM	MEGA-8 (N) 79.0mM	MEGA-9 (N) 25.0mM	MEGA-10 (N) 7.0mM	Mal(11.1) (N) 0.37mM	Mal(11.2) (N) 0.11mM
D	DDAO (N) 10.5mM	n-Decyl-β-D-maltoside (N) 1.8mM	n-Dodecyl-β-D-maltoside (N) 0.2mM	n-Hexadecyl-β-D-maltoside (N) 0.1mM	n-Tetradecyl-β-D-maltoside (N) 0.1mM	n-Tridecyl-β-D-maltoside (N) 0.1mM	n-Undecyl-β-D-maltoside (N) 0.59mM	HECAMEG (N) 19.5mM	IPTG (N) 0.5mM	n-Decanoylsucrose (N) 2.5mM	N-Nonyl-β-D-glucoside (N) 6.5mM	N-Octyl-β-D-glucoside (N) 20.0mM
E	OSGP (N) 9.0mM	Pluronic F-68 (N) 1% w/v	Pluronic F-127 (N) 1% w/v	Sucrose monolaurate (N) 0.3mM	Thesit (N) 0.09mM	Triton X-100 (N) 0.1% v/v	Triton X-114 (N) 0.1% v/v	Nonylphenyl polyethylene glycol (N) 0.1% v/v	Tween 20 (N) 0.1% v/v	Tween 80 (N) 0.1% v/v	GDN (N) 0.018mM	CHAPS (Z) 8.0mM
F	CHAPSO (Z) 8.0mM	BIG CHAP (Z) 2.9mM	Deoxy BIG CHAP (Z) 1.4mM	DDMAB (Z) 4.3mM	LDAO (Z) 2.0mM	Sulfobetaine 8 (Z) 1% w/v	Sulfobetaine 10 (Zwittergent 3-10) (Z) 40mM	Sulfobetaine 12 (Zwittergent 3-12) (Z) 4mM	Sulfobetaine 14 (Zwittergent 3-14) (Z) 0.4mM	Sulfobetaine 16 (Zwittergent 3-16) (Z) 0.06mM	06:0 PC (DHPC) (Z) 15.0mM	07:0 PC (DHPC) (Z) 1.4mM
G	06:0 Lyso PC (Z) 0.5mM	07:0 Lyso PC (Z) 0.5mM	08:0 Lyso PC (Z) 6mM	09:0 Lyso PC (Z) 0.5mM	10:0 Lyso PC (Z) 8.0mM	11:0 Lyso PC (Z) 0.5mM	12:0 Lyso PC (Z) 0.9mM	13:0 Lyso PC (Z) 0.5mM	14:0 Lyso PC (Z) 0.1mM	15:0 Lyso PC (Z) 0.5mM	16:0 Lyso PC (Z) 0.1mM	17:0 Lyso PC (Z) 0.1mM
H	18:0 Lyso PC (Z) 0.1mM	18:1 Lyso PC (Z) 0.1mM	MAPCHO-10 (FOS-Choline-10) (Z) 11.0mM	MAPCHO-12 (FOS-Choline-12) (Z) 1.1mM	MAPCHO-14 (FOS-Choline-14) (Z) 0.12mM	MAPCHO-16 (FOS-Choline-16) (Z) 0.1mM	NDSB-195 (NS) 50mM	NDSB-201 (NS) 50mM	NDSB-211 (NS) 50mM	NDSB-221 (NS) 50mM	NDSB-256 (NS) 50mM	NDSB-256-4T (NS) 50mM

Figure S8. Key for the 96-well detergent screen. This screen from Hampton Research (HR2-407) consisted of 13 ionic (**I**, *wells* **A01-B01**) detergents, 46 non-ionic (N, *wells* **B02-E11**) detergents, 31 zwitterionic detergents (Z, *wells* **E12-H06**), and 6 non-detergent Sulfobetaines (NS, *wells* **H07-H12**). For the ionic detergents, there were 4 cationic (I, *wells* **A05, A06, A07, and A10**), and 9 anionic (I, *wells* **A01-A04, A08, A09, A10-B01**). The final concentrations used in the assay are listed at the bottom of each well, given that each well in the original plate was at a 10x concentration.

A.

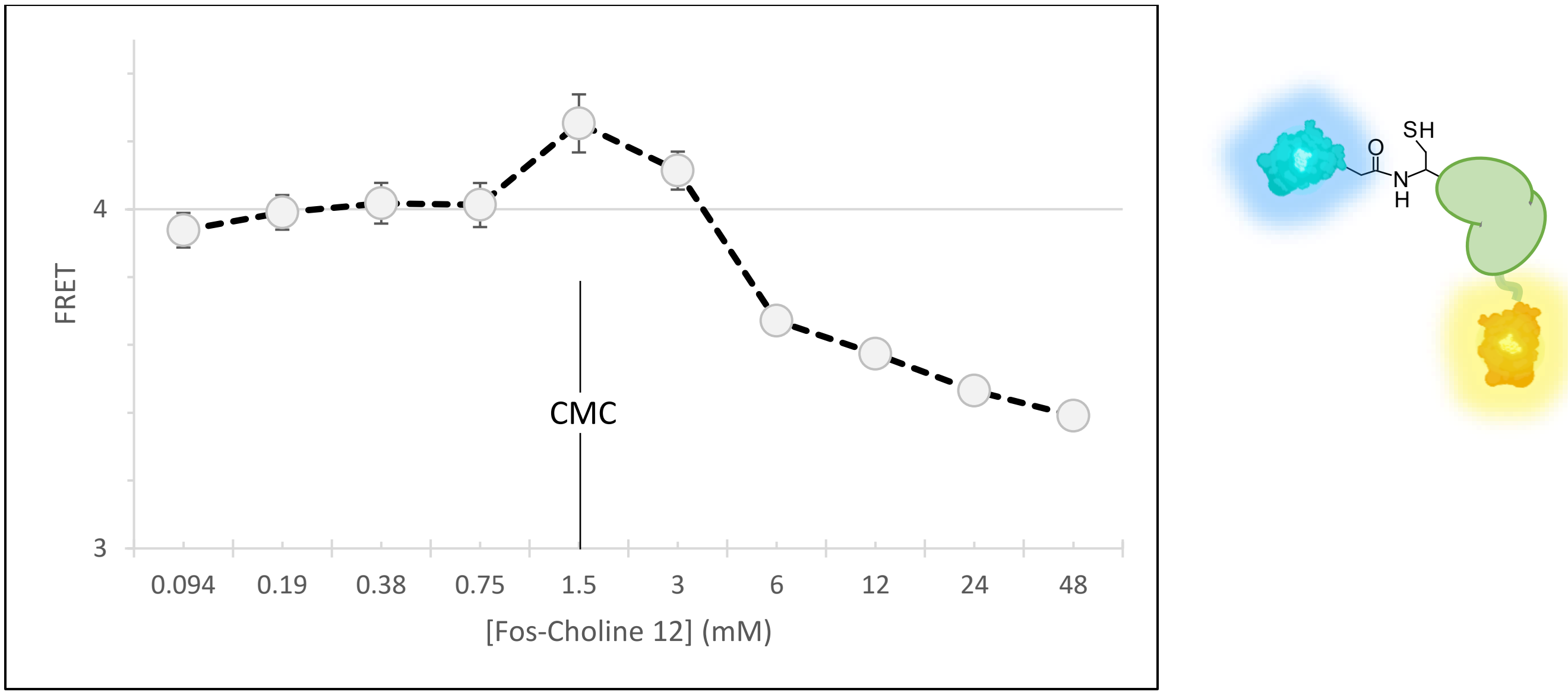

B.

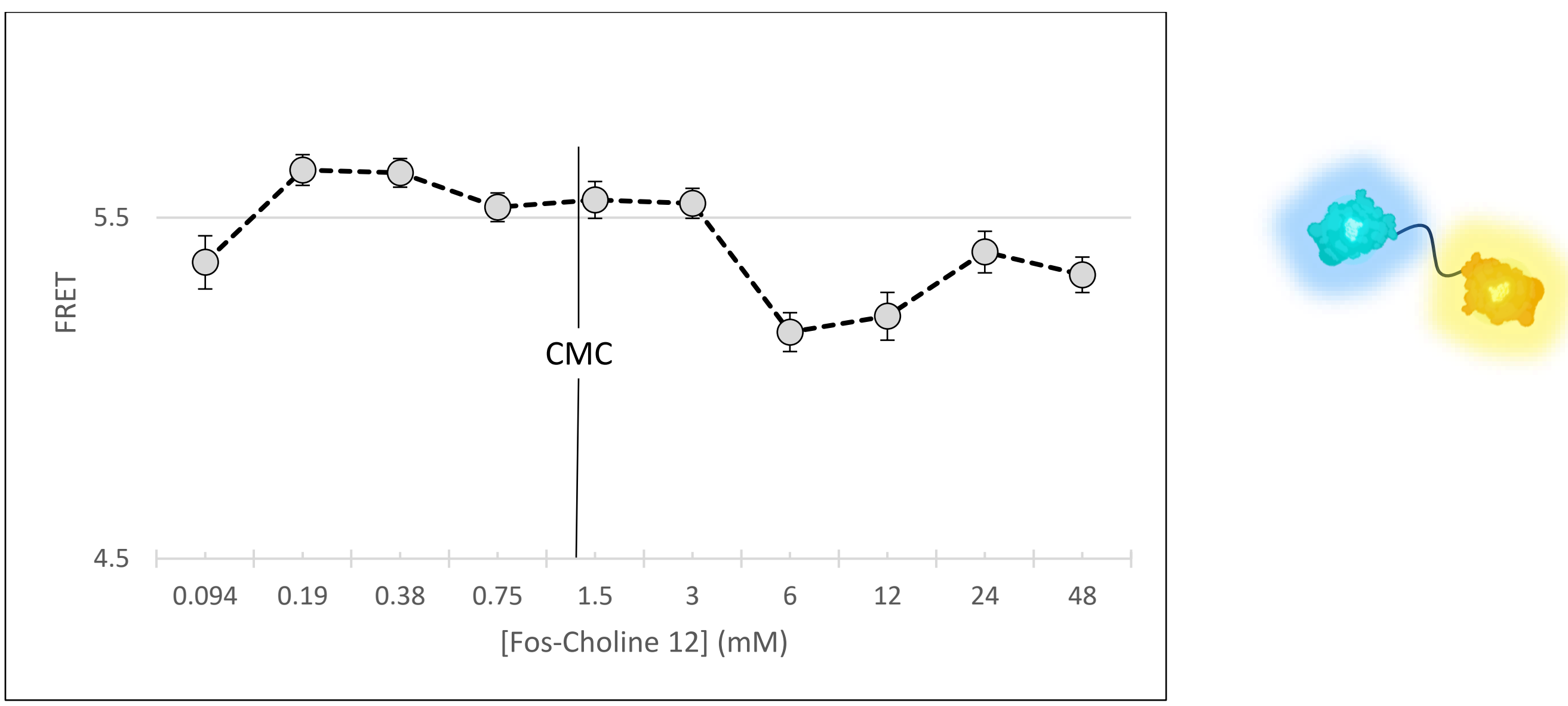

Figure S9. Impact of Fos-Choline 12 concentration on FRET signal from C-H-Y. (*A*) Concentration-response plot of the average FRET for Wild-Type Dme C-H-Y as a function of increasing Fos-Choline 12. (*B*) Concentration-dependent traces representing the average FRET level recorded from control construct, C-Y, at the same Fos-Choline 12 concentration as in (A).

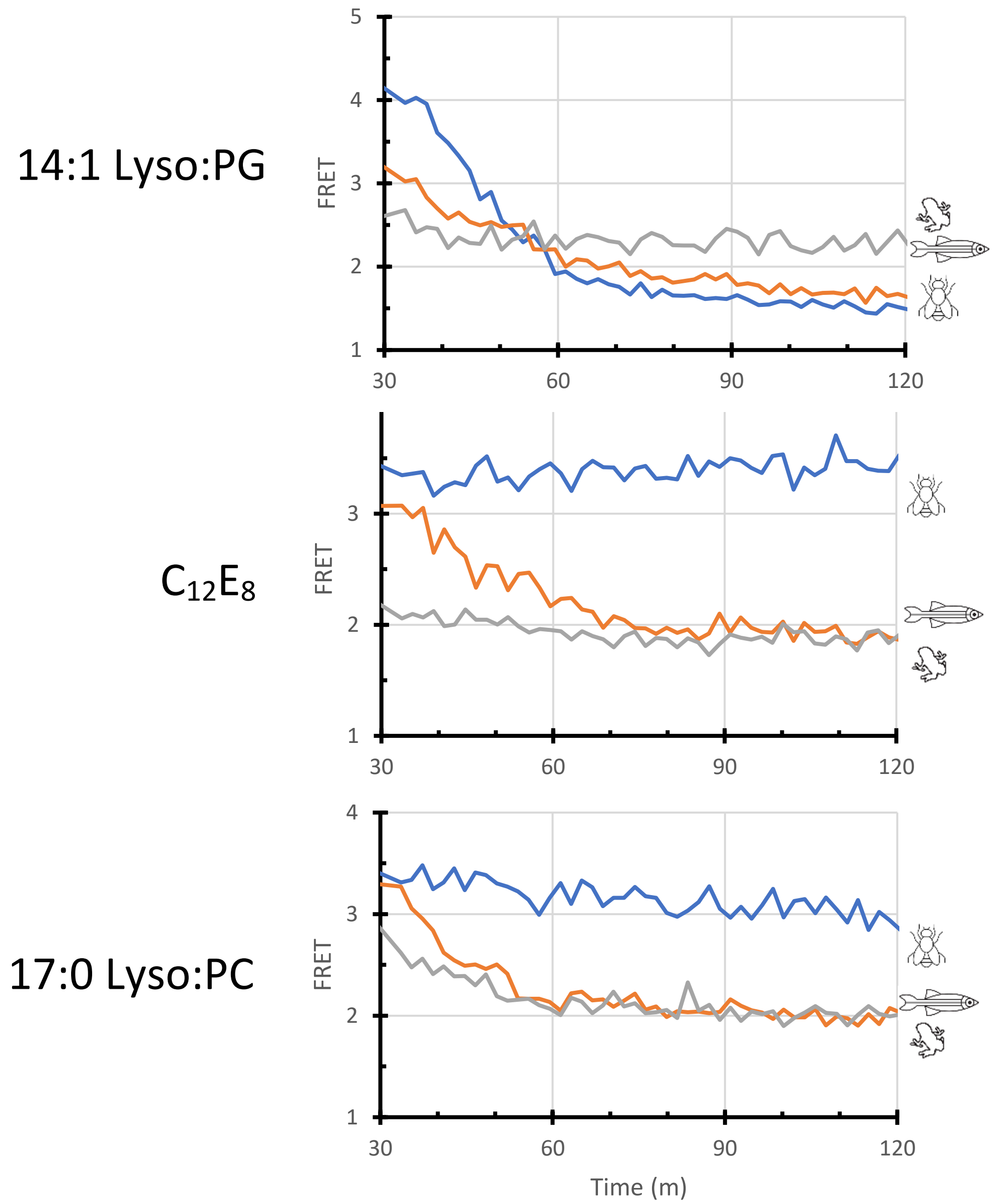

Figure S10. Apparent species-specific detergent effects. Kinetic traces for C-H-Y cholesterolysis using either 14:1 Lyso:PG (*well* **A1**, top), $C_{12}E_8$ (*well* **B9**, middle), or 17:0 Lyso PC (*well* **G12**, bottom) in place of Fos-choline 12. (top) 14:1 Lyso:PG resulted in rapid cholesterolysis for both Dme (blue) and Dre (orange), but no cholesterolysis activity was apparent for Xla (gray). (middle) $C_{12}E_8$ resulted in moderate cholesterolysis for Dre (orange), but no activity for both Dme (blue) and Xla (gray). (bottom) 17:0 Lyso:PC supported rapid cholesterolysis for Xla (gray), moderate cholesterolysis for Dre (orange), and little to no cholesterolysis activity for Dme (blue). Samples were monitored for 2 hours at 30°C in 96-wellplates using 0.2 μM C-H-Y in Bis-Tris buffer (pH 7.1) with EDTA (5 mM), NaCl (0.5 M), with detergent at the respective CMC.

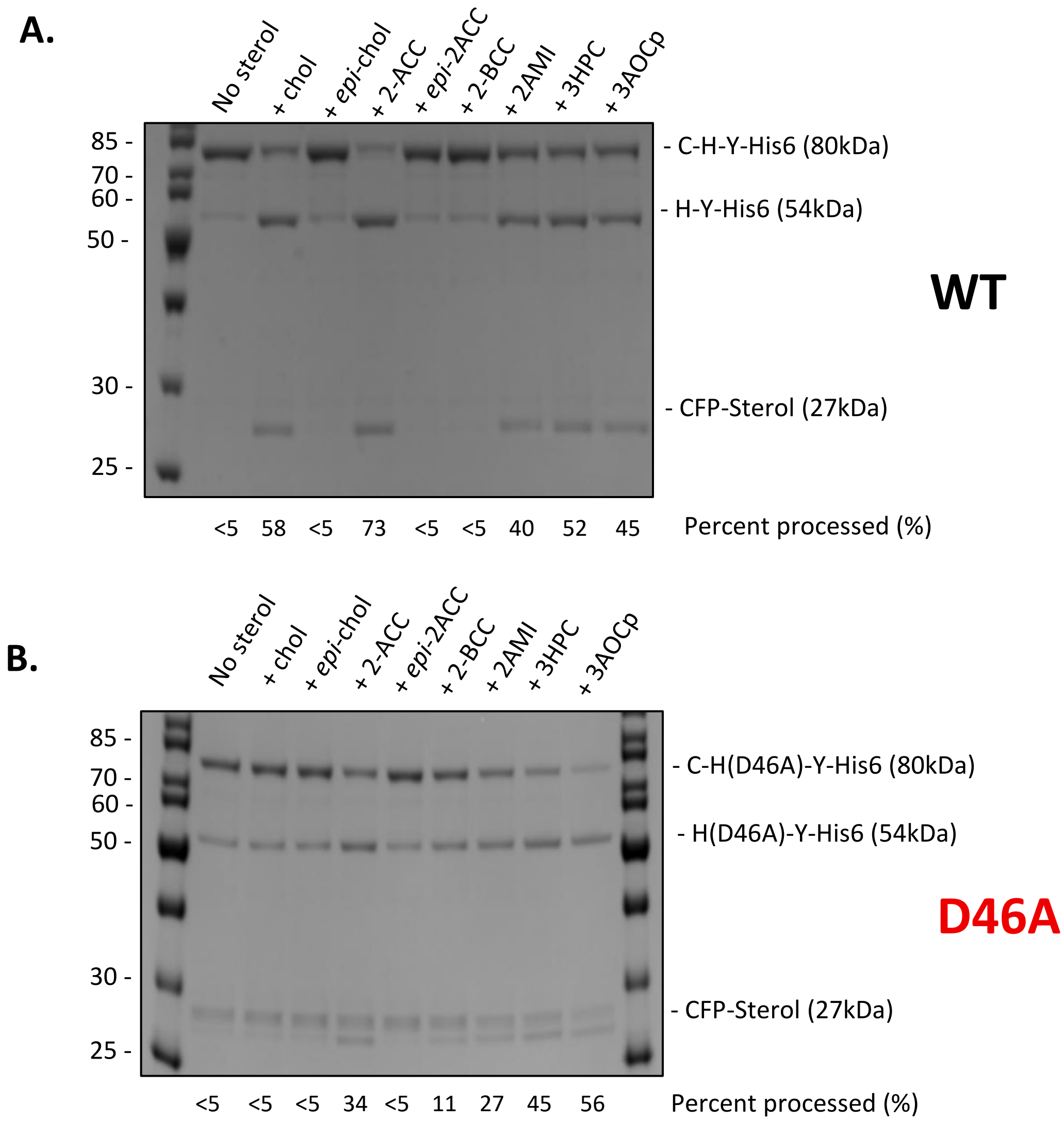

Figure S11. SDS-PAGE Analysis of substrate activity for chemical rescue sterols with Dme Wild-Type C-H-Y and mutant C-H(D46A)-Y. Reactions were run for 3 hours at 30°C using 1 µM protein, Bis-Tris buffer (pH 7.1) also containing EDTA (5mM) and NaCl (0.5 M), and Fos-choline 12 (1.5 mM). (*A*) SDS-PAGE of Dme Wild-Type C-H-Y protein with 50 µM of sterol added for each, except 2-BCC which contained 100 µM of sterol. (B) SDS-PAGE of Dme mutant C-H(D46A)-Y protein with 50 µM of sterol added for each, except 2-BCC which contained 100 µM of sterol. Gels were Coomassie stained for visualization. Percent processed calculations used ImageJ to compare the product bands (C and H-Y) to the total band area (C-H-Y, H-Y, and C). The percent processed (products/total x 100%)) took account of spontaneous hydrolysis by subtracting the % processed in the "no sterol" control sample from each of experimental sample.

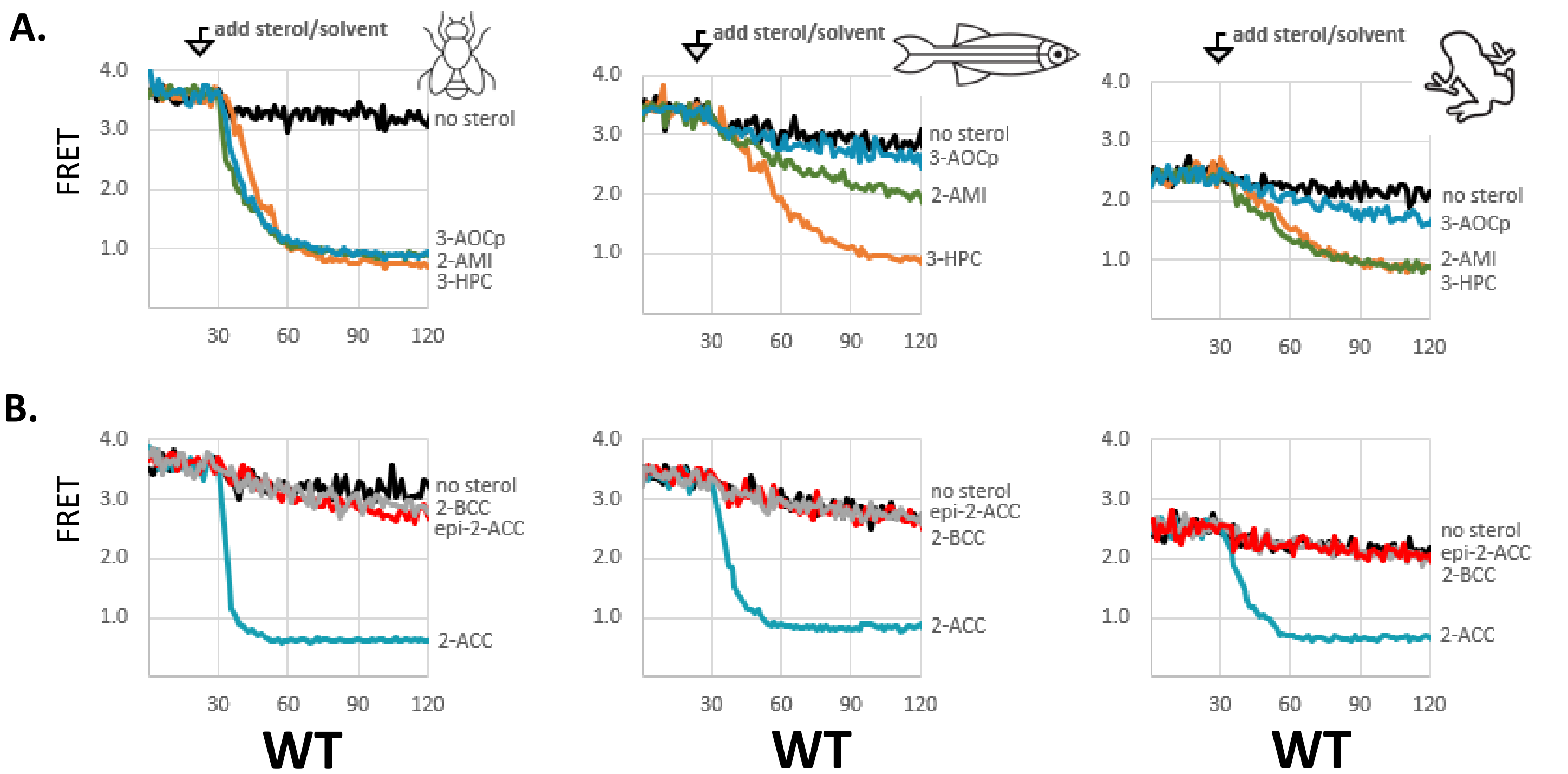

Figure S12. Substrate activity of synthetic sterols using Wild-Type C-H-Y reporter constructs. Samples were monitored for 2 hours at 30°C in 96-wellplates using 0.2 μM C-H-Y in Bis-Tris buffer (pH 7.1) with EDTA (5 mM), NaCl (0.5 M), and Fos-choline 12 (1.5 mM). (*A*) Kinetic traces of Dme (left), Dre (middle), and Xla (right) C-H-Y with either no sterol (black) or with 50 μM 2-AMI (green), 3-HPC (orange), or 3-AOCp (blue). (*B*) Kinetic traces of Dme (left), Dre (middle), and Xla (right) C-H-Y with either no sterol (black) or with 50 μM 2-ACC (cyan), 2-BCC (gray), and *epi* 2-ACC (red).

A.

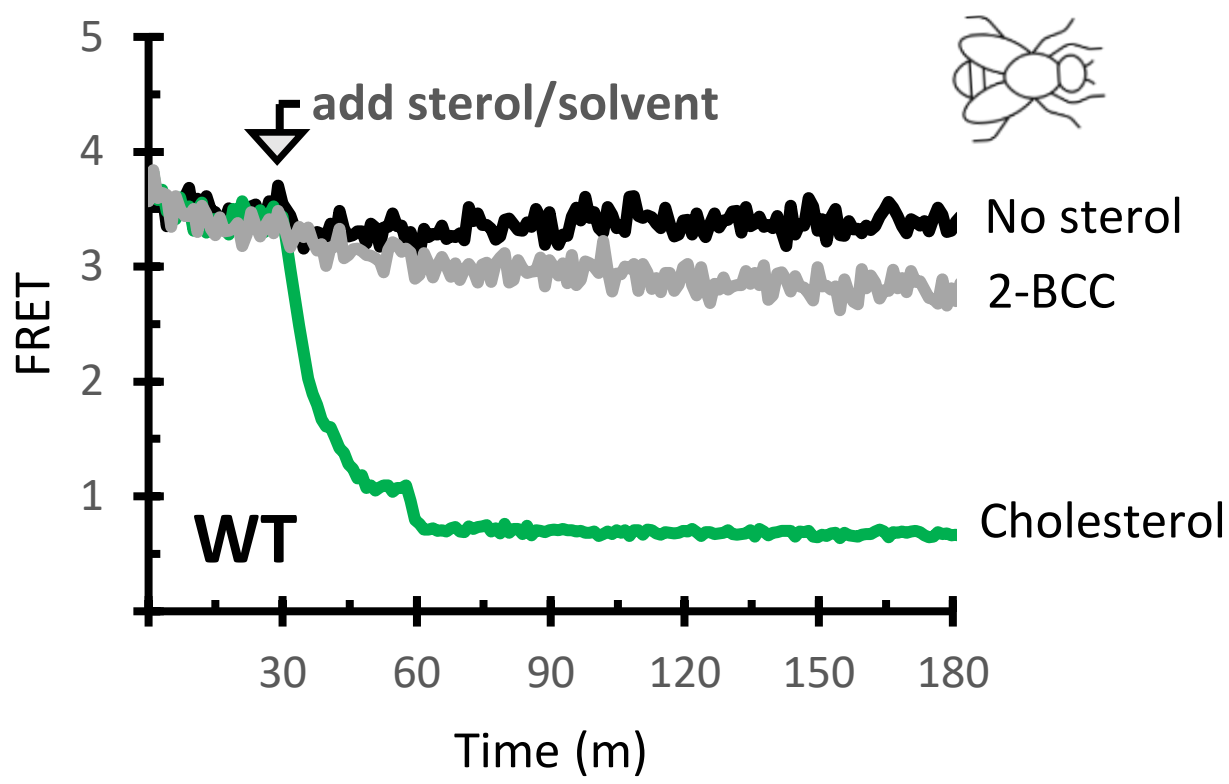

B.

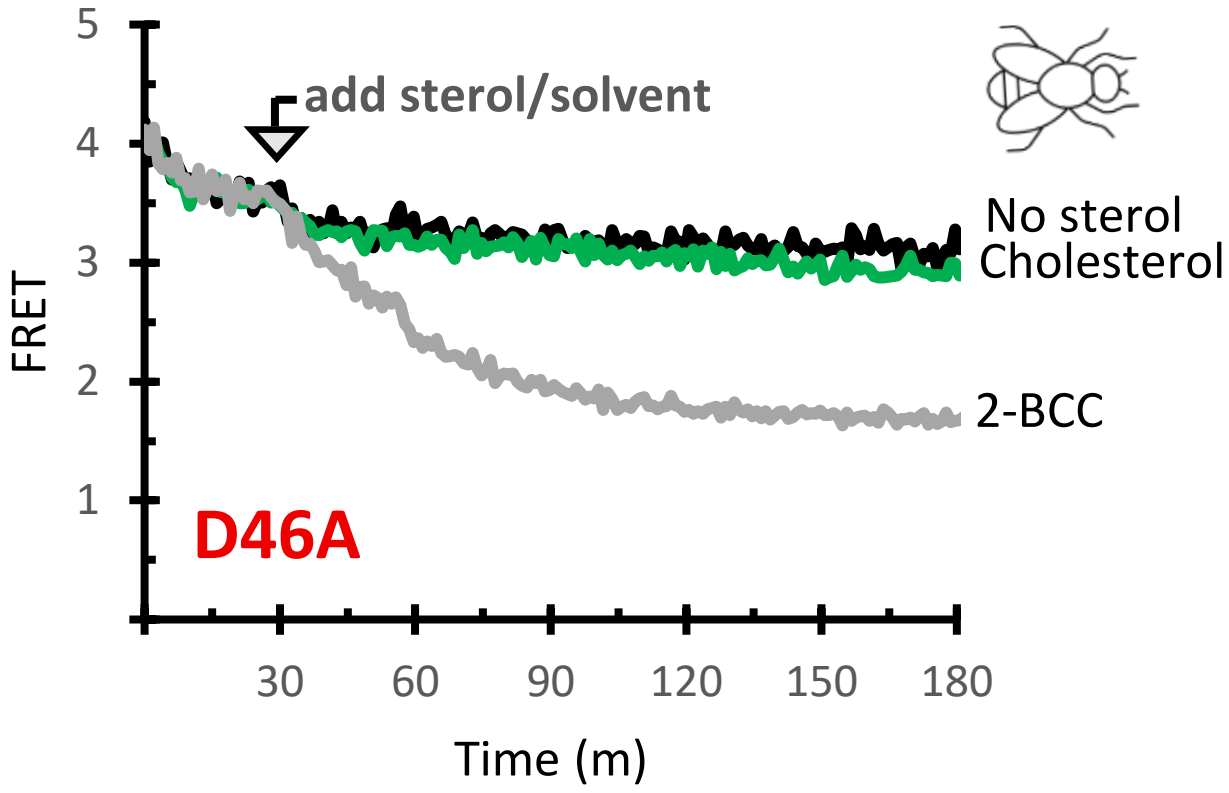

C.

Substrate activity of engineered sterol 2-BCC with Dme Wild-type and D46A mutant HhC[a]

Construct	Sterol	k_{obs} (s^{-1})
Dme HhC (WT)	No sterol	$5.0*10^{-6} \pm 1.6*10^{-6}$
Dme HhC (WT)	Cholesterol	$1.5*10^{-3} \pm 0.2*10^{-3}$
[b] Dme HhC (WT)	2-BCC	$1.9*10^{-5} \pm 0.16*10^{-5}$
[b] Dme HhC (D46A)	No sterol	$1.4*10^{-5} \pm 1.2*10^{-5}$
[b] Dme HhC (D46A)	Cholesterol	$2.0*10^{-6} \pm 0.33*10^{-6}$
Dme HhC (D46A)	2-BCC	$5.0*10^{-4} \pm 0.48*10^{-4}$

[a] Average values reported were calculated using the solver function in Excel with confidence intervals generated using the descriptive statistics function (n=3).

[b] Confidence intervals are estimated based on extrapolated rate constants accrued during a limited period of time that did not allow the reaction to reach completion

Figure S13. Substrate activity of the 2-beta carboxy cholestanol epimer with Dme Wild-Type C-H-Y and mutant C-H(D46A)-Y. Reactions were monitored for 3 hours at 30°C in 96-wellplates using 0.2 µM reporter protein in Bis-Tris buffer (pH 7.1) with EDTA (5 mM) and NaCl (0.5 M), and Fos-choline 12 (1.5 mM). (*A*) Kinetic trace of Dme Wild-Type C-H-Y with either no sterol (black), or with 50 µM cholesterol (light green), or 2-BCC (gray) showed a lack of strong 2-BCC activity. (*B*) Kinetic trace of Dme mutant C-H(D46A)-Y with either no sterol (black), or with 50 µM cholesterol (light green), or 2-BCC (gray). (*C*) Table consisting of the estimated k_{obs} values for the reactions shown in both A and B, calculated using the solver function in Excel. The confidence intervals were generated using the descriptive statistics feature in Excel.

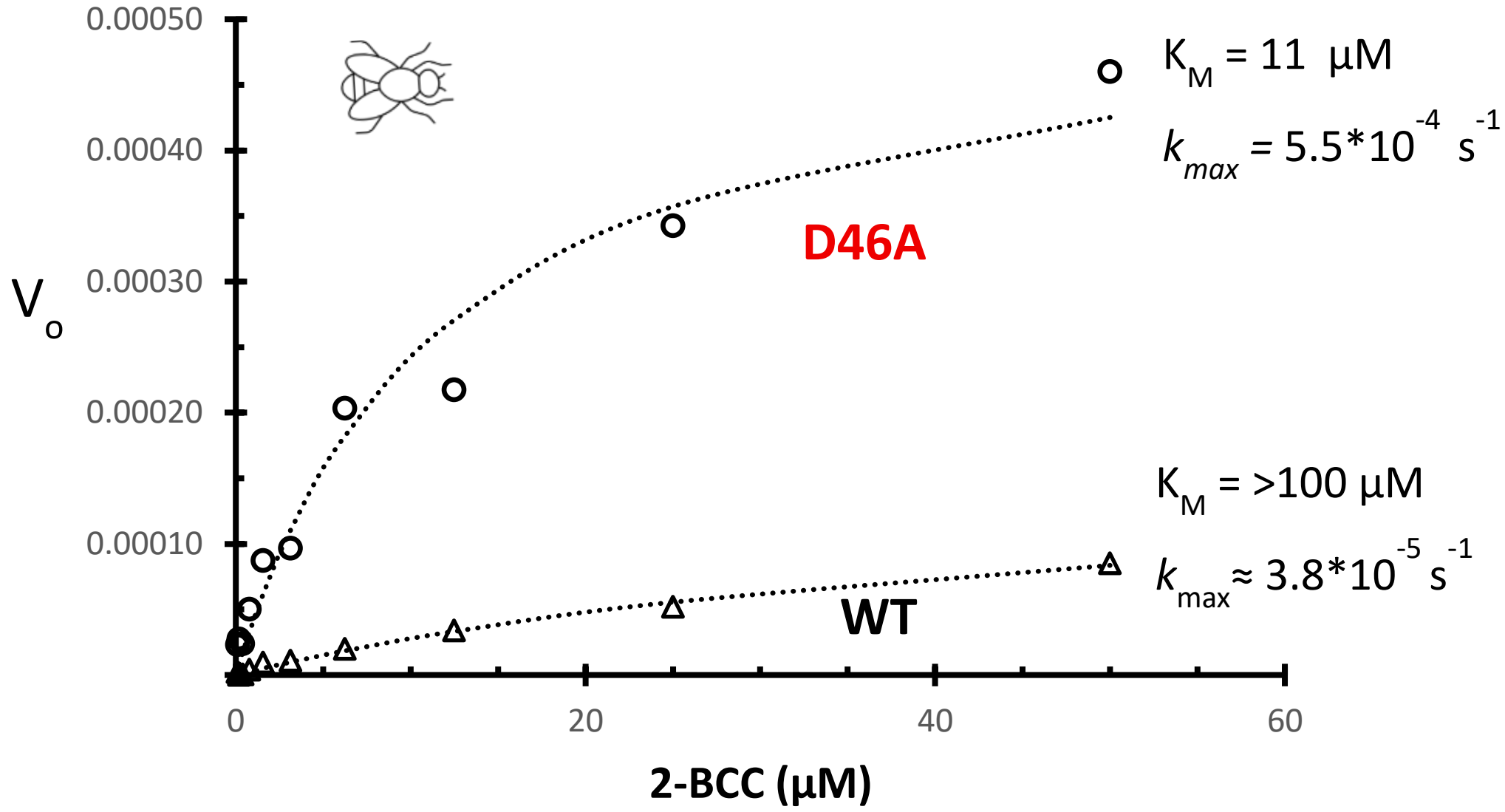

Figure S14. Concentration-dependent substrate activity of the 2-beta carboxy cholestanol epimer with Dme Wild-Type C-H-Y and mutant C-H(D46A)-Y. Reactions were monitored for 2 hours at 30°C in 96-wellplates using 0.2 µM reporter protein in Bis-Tris buffer (pH 7.1) with EDTA (5 mM) and NaCl (0.5 M), and Fos-choline 12 (1.5 mM). Concentration response plot for 2-BCC activity of Dme Wild-Type C-H-Y (Triangle) or C-H(D46A)-Y (Circle) along with an estimated k_{max} and K_M calculated using the solver function in Excel.

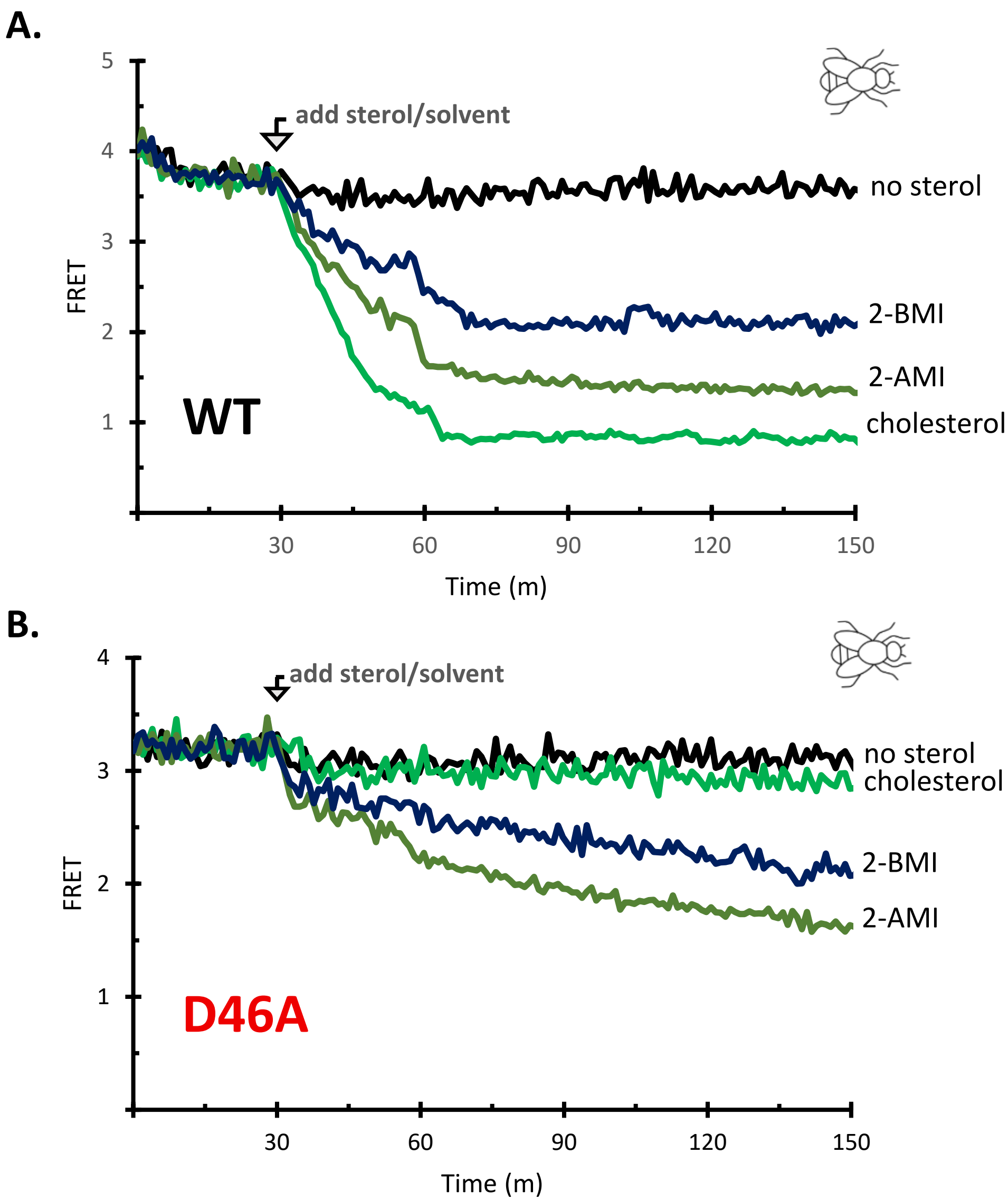

Figure S15. Substrate activity of cholestanol 2-imidazolmethyl epimers with Dme Wild-Type C-H-Y and mutant C-H(D46A)-Y. Reactions were monitored for 2.5 hours at 30°C in 96-wellplates using 0.2 μM reporter protein in Bis-Tris buffer (pH 7.1) with EDTA (5 mM) and NaCl (0.5 M), and Fos-choline 12 (1.5 mM). (*A*) Kinetic trace of Dme Wild-Type C-H-Y with either no sterol (black), or with 50 μM cholesterol (light green), 2-AMI (Dark green), or 2-BMI (Blue). (*B*) Kinetic trace of Dme mutant C-H(D46A)-Y with either no sterol (black), or with 50μM cholesterol (light green), 2-AMI (Dark green), or 2-BMI (Blue).

1. Owen, T. S.; Ngoje, G.; Lageman, T. J.; Bordeau, B. M.; Belfort, M.; Callahan, B. P., Forster resonance energy transfer-based cholesterolysis assay identifies a novel hedgehog inhibitor. *Anal Biochem* **2015,** *488*, 1-5.
2. Owen, T. S.; Xie, X. J.; Laraway, B.; Ngoje, G.; Wang, C.; Callahan, B. P., Active site targeting of hedgehog precursor protein with phenylarsine oxide. *Chembiochem* **2015,** *16* (1), 55-8.
3. Smith, C. J.; Wagner, A. G.; Stagnitta, R. T.; Xu, Z.; Pezzullo, J. L.; Giner, J. L.; Xie, J.; Covey, D. F.; Wang, C.; Callahan, B. P., Subverting Hedgehog Protein Autoprocessing by Chemical Induction of Paracatalysis. *Biochemistry* **2020,** *59* (6), 736-741.
4. Wagner, A. G.; Stagnitta, R. T.; Xu, Z.; Pezzullo, J. L.; Kandel, N.; Giner, J. L.; Covey, D. F.; Wang, C.; Callahan, B. P., Nanomolar, Noncovalent Antagonism of Hedgehog Cholesterolysis: Exception to the "Irreversibility Rule" for Protein Autoprocessing Inhibition. *Biochemistry* **2022,** *61* (11), 1022-1028.
5. Childs, M. E.; Weber, W. P., Preparation of Cyanoformates - Crown Ether Phase Transfer Catalysis. *J Org Chem* **1976,** *41* (21), 3486-3487.
